# Unusual Presentation of Nevus Lipomatosus Cutaneous Superficialis: A Rare Location of a Rare Diagnosis

**DOI:** 10.7759/cureus.60902

**Published:** 2024-05-23

**Authors:** Debaleena Ghosh, Disha Chakraborty, Shailaja Verma, Abhishek De

**Affiliations:** 1 Dermatology, Calcutta National Medical College and Hospital, Kolkata, IND

**Keywords:** hamartoma, papules, tumors, differentials, skin cancer, nevus lipomatosus cutaneous superficialis, nose, nevus

## Abstract

The nose is a common site for many dermatological disorders and even skin cancers. Herein, we report a case of an elderly man who presented with papular lesions on his nose. A 64-year-old man presented with a cluster of four to five skin-colored papules on his nose for the last two years which were gradually increasing in size. He also had rhinophyma for the past 10 years. Routine investigations and histopathological examination were performed. On biopsy, it was revealed to be nevus lipomatosus cutaneous superficialis, a rare, benign hamartomatous anomaly found mostly in lower parts of the body like the buttocks and hence not usually considered a differential in such cases. It is essential to know about this rare entity as well as its atypical features to consider it as a differential diagnosis for such lesions on the nose.

## Introduction

Nevus lipomatosus cutaneous superficialis (NLCS) is a rare, benign hamartomatous lesion, first described by Hoffman and Zurhelle [1]. It is characterized clinically by skin-colored or yellowish soft papules with a smooth or wrinkled surface, which are histologically composed of groups of mature fat cells located among the bundles of dermal collagen [2]. It is mostly of two types - classical and solitary. Most cases of NLCS are found in the lower parts of the body like the buttocks, thighs, and flanks [3]. Here we are reporting a case of nevus lipomatosus where an elderly man presented with papular lesions on the nose.

## Case presentation

A 64-year-old male presented with a cluster of four to five skin-colored papules that had been present on the right half of his nose for two years. These papules were gradually increasing in size. Although there was occasional bleeding, there were no other symptoms. The lesions were preceded by rhinophyma for the past 10 years.

**Figure 1:**
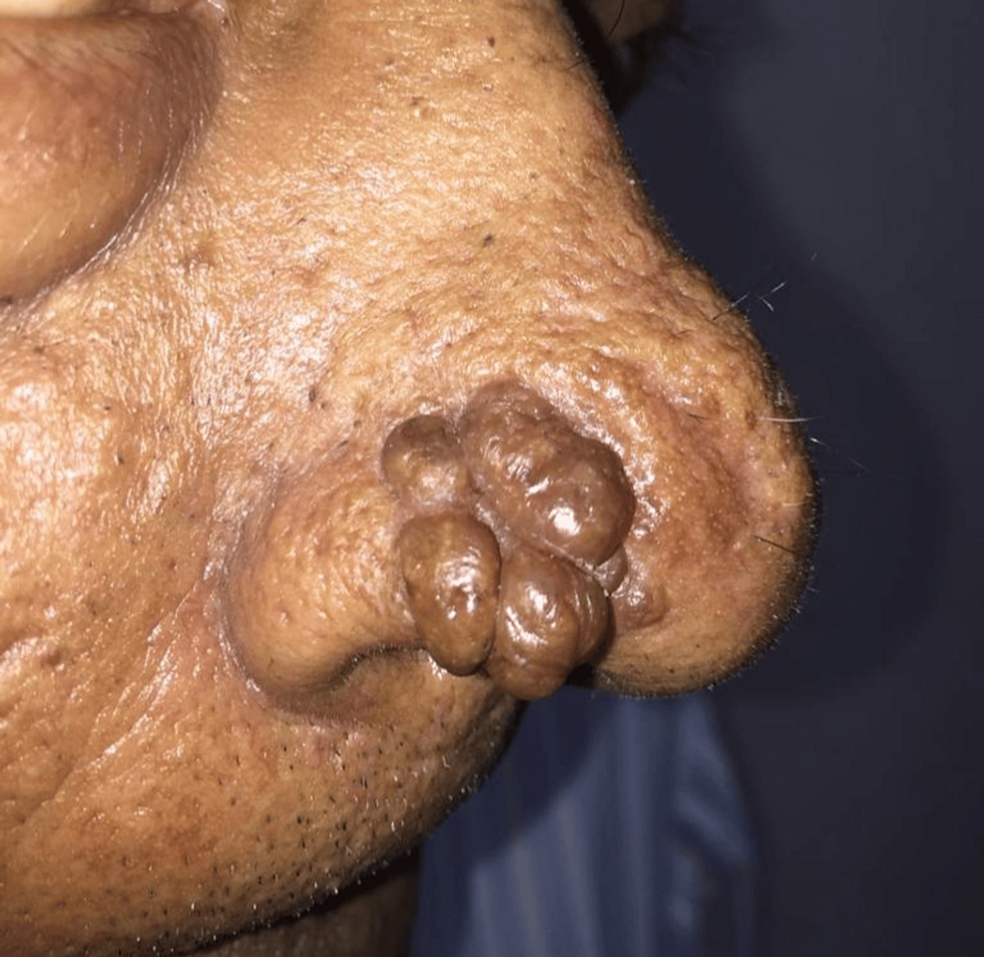
Soft, sessile, non-tender, non-ulcerated papules with an uneven surface were found in a flower-like arrangement on the right half of the nose, covering an area of 2cm×2cm.

On cutaneous examination, soft, sessile, non-tender, non-ulcerated papules with an uneven surface were found in a flower-like arrangement on the right half of the nose, covering an area of 2 cm × 2 cm (Figure 1).

On closer examination, we found that the surface had dilated blood vessels and comedo-like openings in a few of the papules.

**Figure 2:**
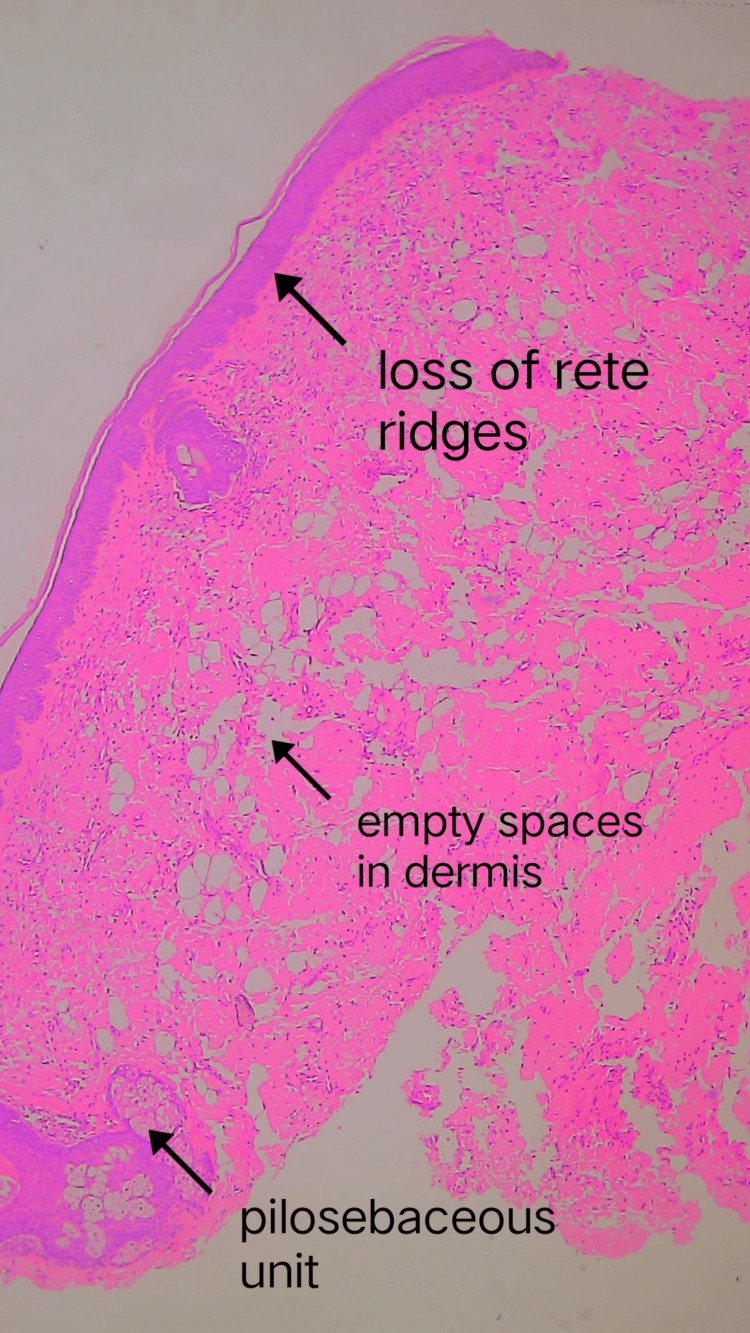
Hematoxylin and eosin staining (4X) showing loss of rete ridges and ectopic mature adipocytes among collagen fibers in the dermis

**Figure 3:**
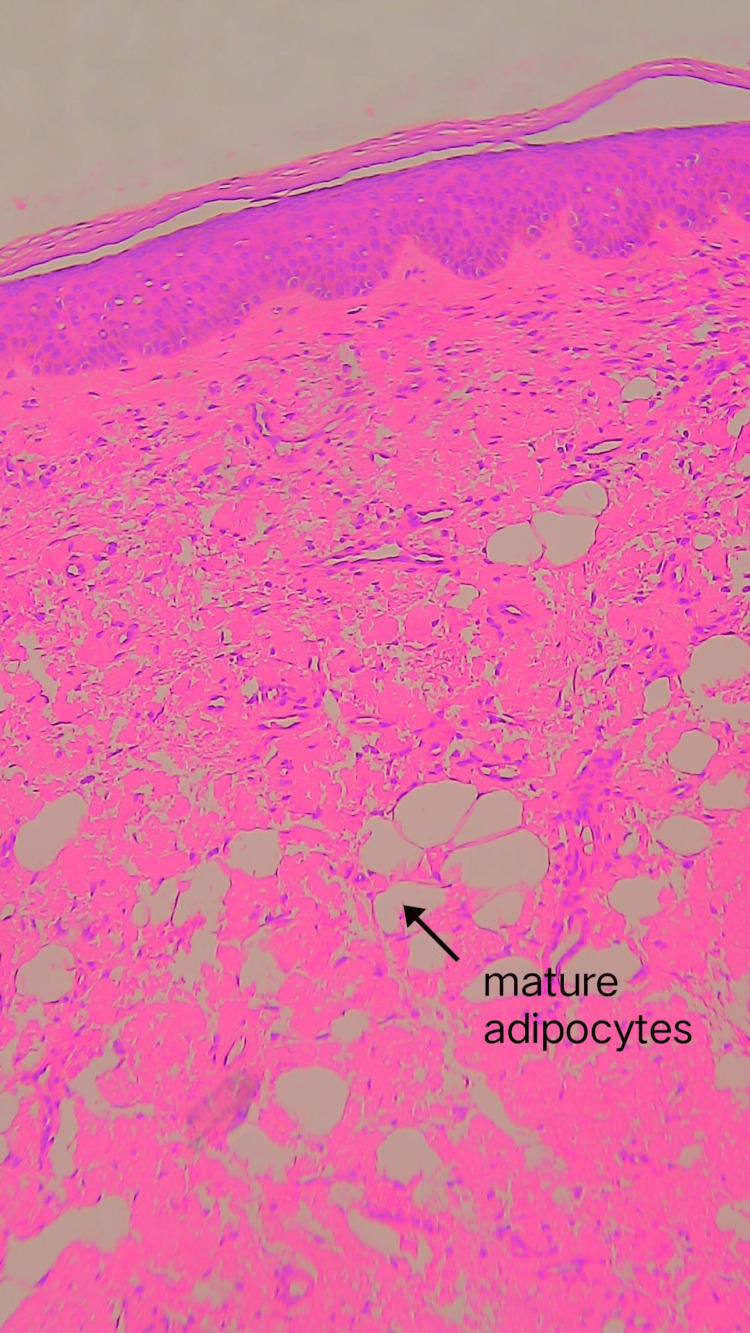
Hematoxylin and eosin staining (10X) showing similar features.

All routine blood investigations were within normal limits. Differentials considered prior to biopsy were trichoepithelioma, angiolymphoid hyperplasia with eosinophilia, cutaneous sarcoidosis, and papular xanthoma. A few other differentials were focal dermal hypoplasia or Goltz syndrome and lipofibroma. We were surprised when the histopathological examination with hematoxylin and eosin staining revealed loss of rete ridges and ectopic mature adipocytes among collagen fibers in the dermis (Figures 2-3).

**Figure 4:**
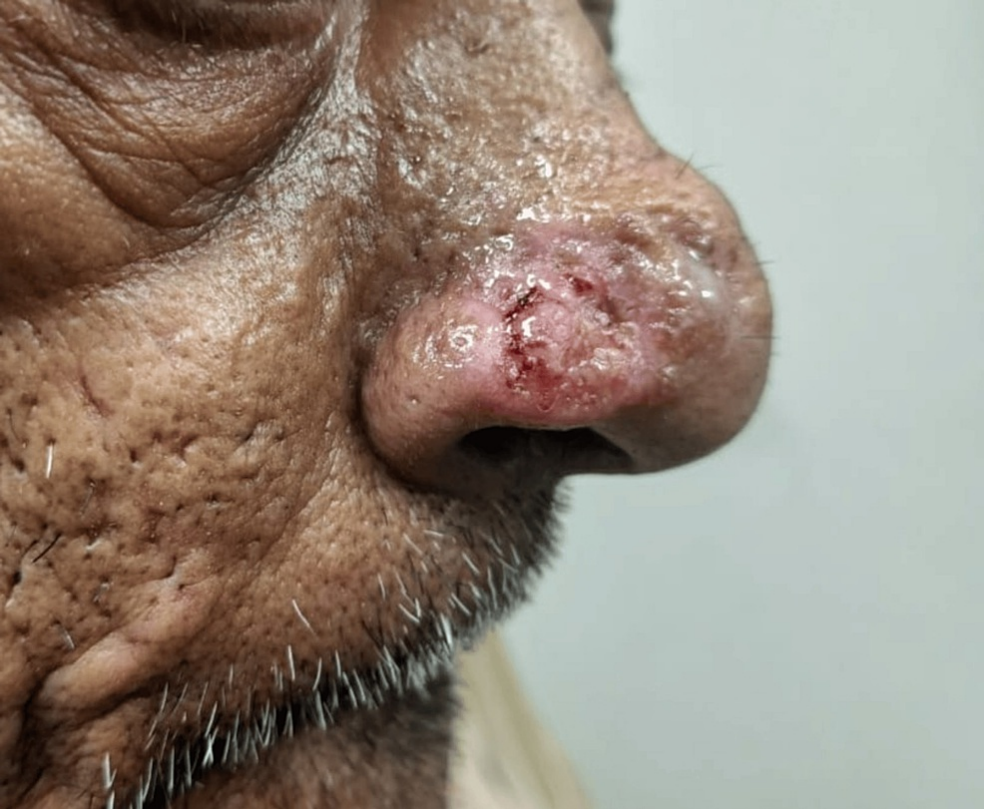
Image of the patient after treatment with radiofrequency ablation

A diagnosis of NLCS (classical type) was made. We treated the lesions with radiofrequency ablation and on a follow-up after six months, no recurrence was noted (Figure 4). Other conditions that are closely associated with it are appendageal tumors like cylindroma, nevus sebaceous, and fibrofolliculoma. However, our histopathology findings clearly pointed in favor of NLCS.

## Discussion

Nevus lipomatosus, described by Hoffman and Zurhelle in 1921, is an uncommon hamartoma primarily consisting of two types - classical or multiple types. The former is more common and occurs mostly at birth, where multiple soft, elastic, skin-colored, cerebriform, and pedunculated papules or nodules can coalesce to form plaques. This is mostly found in the gluteal region, thighs, and pelvis [3]. The rarer solitary form, which appears after the second decade of life, is characterized as sessile, dome-shaped nodules or papules that appear in variable locations, but with a predilection for the trunk area [4]. Other atypical locations like the neck, scalp, eyelids, clitoris, and groin area were also reported [5-7]. Histopathological analysis plays a crucial role in diagnosing NLCS. A defining feature seen under the microscope is the presence of adipose tissue in the papillary and/or reticular dermis. Additionally, the overlying epidermis may exhibit hyperkeratosis, acanthosis, papillomatosis, and focal elongation of rete ridges, while adnexal structures may appear either normal or attenuated [6]. NLCS is typically asymptomatic, as seen in the current case. However, there have been rare instances where ulceration has been noted following external trauma or ischemia. Some alterations like hypertrichosis, comedo-like openings, café-au-lait macules, and leucodermic spots can sometimes coexist with NLCS. Similarly, the nevus surface displayed multiple comedo-like openings in our case. Some authors have reported NLCS alongside other cutaneous conditions like follicular papules, hemangiomas, and angiokeratoma of Fordyce [7].

Although not much is known about etiopathogenesis, 2p24 deletion might be associated with it. NLCS was previously believed to be caused by degenerative changes in connective tissues. However, the exact cause of fat deposition in the dermis in NLCS is still unknown. Studies have not supported this theory, suggesting instead that fat cells may develop locally as heterotopic adipose tissue. NLCS is thought to occur due to the displacement of subcutaneous adipose tissue embedded in the dermis. Recent electron microscopic studies have strongly supported the perivascular origin of young adipocytes and their subsequent maturation into mature fat cells [8].

NLCS itself is an uncommon case. Moreover, classical-type NLCS on the nose has been reported once to date [9]. Ours is the second case reported, to the best of our knowledge. Another unique finding in our patient was the late onset of lesions (sixth decade of life) which is unlikely for classical NLCS where lesions are usually congenital or start appearing within the first decade of life.

## Conclusions

The nose is a common site for many dermatological disorders and even cutaneous malignancies, especially in older age groups. Given its atypical presentation, this case highlights the importance of considering NLCS in the differential diagnosis of lesions presenting in the nose, despite its rarity in this location. Clinicians should be aware of this entity to ensure accurate diagnosis and appropriate management of similar cases in the future.
